# Critically ill children with SARS-COV-2 Omicron infection at a national children medical center, Guangdong, China

**DOI:** 10.1186/s12887-024-04735-w

**Published:** 2024-04-15

**Authors:** Fen Lin, Dao-Ju Jiang, Song Zhang, Zhe Yang, Hua-Song Zeng, Zhi-Ping Liu, Li-Ye Yang

**Affiliations:** 1 Precision Medical Lab Center, Chaozhou Central Hospital, Chaozhou, Guangdong Province P. R. China; 2 Department of Allergy, Immunology and Rheumatology, Guangzhou Women and Children’s Medical Center, Guangdong Province, P. R. China; 3 Department of Pediatrics, Chaozhou Central Hospital, Chaozhou, Guangdong Province P. R. China; 4 Precision Medical Lab Center, People’s Hospital of Yangjiang, No.42 Dongshan Road, Jiangcheng District, Yangjiang, 529500 Guangdong Province P. R. China

**Keywords:** Critical ill children, SARS-CoV-2, Omicron variant, Bacterial co-infection, Nervous system

## Abstract

**Background:**

SARS-CoV-2 infection is described as asymptomatic, mild, or moderate disease in most children. SARS-CoV-2 infection related death in children and adolescents is rare according to the current reports. COVID-19 cases increased significantly in China during the omicron surge, clinical data regarding pediatric critical patients infected with the omicron variant is limited. In this study, we aim to provide an overview of the clinical characteristics and outcomes of critically ill children admitted to a national children’s medical center in Guangdong Province, China, during the outbreak of the omicron variant infection.

**Methods:**

We conducted a retrospective study from November 25, 2022, to February 8, 2023, which included 63 critically ill children, under the age of 18, diagnosed with SARS-CoV-2 infection. The patients were referred from medical institutions of Guangdong province. The medical records of these patients were analyzed and summarized.

**Results:**

The median age of patients was 2 years (Interquartile Range, IQR: 1.0–8.0), sex-ratio (male/female) was 1.52. 12 (19%) patients (age ≥ 3 years) were vaccinated. The median length of hospital stay was 14 days (IQR: 6.5–23) in 63 cases, and duration of fever was 5 days (IQR: 3-8.5), pediatric intensive care unit (PICU) stay was 8 days (IQR 4.0–14.0) in 57 cases. 30 (48%) cases had clear contact history with family members who were infected with SARS-CoV-2. Three children who tested positive for SARS-CoV-2 infection did not show any abnormalities on chest imaging examination. Out of the total patients, 33 (52%) had a bacterial co-infection, with *Staphylococcus aureus* being the most commonly detected bacterial pathogen. Our cohort exhibited respiratory and nervous system involvement as the primary features. Furthermore, fifty (79%) patients required mechanical ventilation, with a median duration of 7 days (IQR 3.75–13.0). Among these patients, 35 (56%) developed respiratory failure, 16 (25%) patients experienced a deteriorating progression of symptoms and ultimately succumbed to the illness, septic shock was the most common condition among these patients (15 cases), followed by multiple organ failure in 12 cases, and encephalopathy identified in 7 cases.

**Conclusion:**

We present a case series of critically ill children infected with the SARS-CoV-2 omicron variant. While there is evidence suggesting that Omicron may cause less severe symptoms, it is important to continue striving for measures that can minimize the pathogenic impact of SARS-CoV-2 infection in children.

**Supplementary Information:**

The online version contains supplementary material available at 10.1186/s12887-024-04735-w.

## Introduction

The emergence of the SARS-CoV-2 Omicron variant (B.1.1.529) in South Africa on November 9, 2021 marked the onset of a rapid global spread of the virus. The Omicron genome has given rise to a distinct monophyletic lineage, distinguished by the presence of over 30 mutations in the spike protein. As a result, the virus exhibits heightened affinity for human cells, leading to increased infection rates and evasion of the immune response [1, 2]. Many studies reported the lower death and ICU admission rates among Omicron infected patients compared to the other variants [3, 4]. However, while the severity of infections in children hospitalized during the Omicron wave may have been lower, there still existed a very real and substantial risk for severe illness [5, 6]. For example, one study of China revealed that nine children (1.37%) died among 659 hospitalized children with COVID-19 during the Omicron era, and five cases were diagnosed with acute necrotizing encephalopathy (ANE) [5].

China implemented ‘zero COVID’ strategies since August 2021 to combat SARS-CoV-2 infections. Chinese government has continuously adjusted and optimized epidemic prevention and control policies to reflect the evolving situation in 2022. With gradual relaxation of policies, the number of individuals infected with SARS-CoV-2 has continued to rise. Furthermore, despite the government’s efforts to promote widespread vaccine accessibility activities starting in July 2021, which notably boosted vaccination rates among vulnerable demographics like the elderly, ethnic minorities, and individuals with disabilities, a significant portion of the population remains unvaccinated. By the end of 2022, a massive SARS-CoV-2 Omicron wave spread through the country [5–8]. The significant surge of COVID-19 in China can be attributed not only to the transmissibility of the SARS-CoV-2 Omicron variant, but also to the population’s lack of herd immunity and the suboptimal protection provided by the vaccines. During this wave, a surge of hospitalized patients has strained healthcare systems, although mortality rates have been relatively lower [5, 6]. Particularly noteworthy is the significant rise in hospitalizations of children infected with the Omicron variant during this period [5, 6, 9, 10]. While severe illness from COVID-19 is rare in pediatric patients, most cases in children present milder symptoms compared to infected adults, such as fever, cough, rhinorrhea, diarrhea, nausea/vomiting, or an asymptomatic state [11–13].

From December 8, 2022, to January 2023, an epidemic of the Omicron strain also occurred in Guangdong, China. There was only a few reports on Omicron variant infections of severe or critical cases among children following this outbreak [5, 6]. Guangzhou Women and Children’s Medical Center, a national tertiary hospital for children in Guangdong Province, China, has been actively treating a large number of sick children, including referrals from other medical institutions. In this report, we present a series of critical cases of children with SARS-CoV-2 Omicron infection, which will contribute to our understanding of the severe form of COVID-19 in children.

## Methods

### Study design and patients

We conducted a retrospective study at a national children’s medical center in Guangdong, China, between November 25, 2022, and February 8, 2023. The study encompassed all children under the age of 18 who were critically ill and diagnosed with COVID-19. These children tested positive for SARS-CoV-2 nucleic acid in nasal and/or oropharyngeal swabs, and their diagnoses were supported by a combination of clinical manifestations and imaging findings observed during their hospitalization. We followed the disease severity classification provided by the World Health Organization (WHO) and the “New coronavirus pneumonia diagnosis and treatment protocol (Trial Version 10)” issued by the China National Health Commission to determine the severity of the cases [14, 15]. In summary, critical cases were defined as those meeting at least one of the major criteria, which include patients requiring mechanical ventilation for respiratory failure, patients requiring vasoactive medications for septic shock, or patients experiencing other organ failure that necessitates monitoring and treatment in the intensive care unit (ICU). Moreover, patient files were reviewed to identify cases of multisystem inflammatory syndrome in children (MIS-C) based on the diagnostic criteria of WHO [16]. The criteria were as follows: (1) Children and adolescents 0–19 years of age with fever > 3 days. And two of the following: Rash or bilateral non-purulent conjunctivitis or muco-cutaneous inflammation signs (oral, hands or feet); Hypotension or shock. Features of myocardial dysfunction, pericarditis, valvulitis, or coronary abnormalities (including ECHO findings or elevated Troponin/NT-proBNP); Evidence of coagulopathy (by prothrombin time (PT), activated partial thromboplastin time (APTT), elevated D-dimers); Acute gastrointestinal problems (diarrhoea, vomiting, or abdominal pain). (2) Elevated markers of inflammation such as erythrocyte sedimentation rate (ESR), C-reactive protein, or procalcitonin (PCT). (3) No other obvious microbial cause of inflammation, including bacterial sepsis, staphylococcal or streptococcal shock syndromes. (4) Evidence of COVID-19 antigen test or serology positive.

### Data collection

We conducted a comprehensive review of the medical records for all critically ill children who were admitted to the hospital during the study period and diagnosed with confirmed SARS-CoV-2 infection using reverse transcription polymerase chain reaction (RT-PCR) testing. The patients’ data encompassed various aspects, including demographic information, medical history, symptoms and signs, complications, laboratory findings, radiology examinations, treatment interventions, the need for ventilator support, and occurrence of adverse events. The clinical outcomes of interest were classified as either cured/discharge or mortality. Additionally, we also documented the vaccination status of the patients.

### Laboratory confirmation

We performed RNA extraction on nasal swab and/or oropharyngeal swab samples, followed by testing for the presence of SARS-CoV-2 viral RNA using an in-house Taqman RT-real-time PCR assay. The assay specifically targeted the *N* and *ORF1ab* genes, in accordance with the manufacturer’s protocol [17]. PCR testing for other respiratory viruses (Flu virus A and B, parainfluenza virus, respiratory syncytial virus, rhinovirus, adenovirus, epstein-barr virus and bocavirus) besides SARS-CoV-2 was indicated to identify co-infections or alternative causes of respiratory illness in children with COVID-19. Metagenomic next-generation sequencing (mNGS) was used to identify other potential pathogens causing respiratory symptoms when the diagnosis was unclear or there were atypical presentations.

### Discharge criteria

The clinical condition was stable and the symptoms and signs disappeared.

### Data analysis

All statistical analyses were conducted using SPSS 21.0 software. Descriptive statistics were employed to summarize all variables. Categorical data were expressed as number (n) and percentages (%), while continuous data were presented as either means and standard errors or medians and interquartile range (IQR).

### Ethical considerations

This study adhered to the ethical guidelines outlined in the Declaration of Helsinki and was approved by both the Institutional Review Board of Guangzhou Women and Children’s Medical Center (No.81,801,905) and Chaozhou Central Hospital (No.2,023,016). Given the retrospective nature of the study, the Ethics Committee of Guangzhou Women and Children Medical Center waived the need for the informed consent.

## Results

### Demographic data and clinical characteristics

From November 25, 2022, to February 8, 2023, a total of 702 children who tested positive for SARS-CoV-2 PCR were admitted into our hospital, and 63 cases were identified as critically ill, and 57 cases required intensive care at the PICU, the primary reasons for PICU admission were respiratory failure (31 cases), shock (7 cases), acute encephalopathy (6 cases), status epilepticus (5 cases), multiple organ failure (3 cases), cardiopulmonary arrest (3 cases), hepatic failure (1 case), renal failure (1 case), and intestinal hernia with bowel obstruction (1 case). Among 63 children, the median age was 2 years (IQR: 1.0–8.0) and the sex ratio was 1.52 (38 males/25 females). 61.9% (39/63) of patients were under 3 years old, twelve of them had received one or two doses of the COVID-19 vaccine (China-made inactivated CoronaVac, including Sinopharm and Sinovac).

The median length of hospital stay for 63 patients was 14 days (IQR: 6.5–23), and the median duration of fever was 5 days (IQR: 3-8.5). PICU stay was 8 days (IQR 4.0–14.0) in 57 cases. 30 (48%) cases had a positive contact history with a family member who had been infected with SARS-CoV-2. Additionally, 9 children had underlying diseases, with 6 of them having epilepsy, one with nephrotic syndrome (one year history of nephrotic syndrome, 24 mg/day of methylprednisolone orally for immunosuppression) and one with cerebral palsy. Furthermore, one patient had acute myeloid leukemia and had undergone hematopoietic stem cell transplantation 40 months ago. At the time of admission, she was 17 years old, experiencing chronic GVHD, and had not been on any immunosuppressive agents prior to admission.

All patients in our cohort experienced at least one complication. The most prevalent complications were respiratory failure (35 cases, 55.6%) and encephalopathy (44 cases, 69.8%). Comparison between patients with and without neurological complications was shown in Table S1. Septic shock and epilepsy occurred in 22 (34.9%) and 10 (15.9%) patients, respectively. During hospitalization, 38 (60%) critical ill patients developed MIS. Unfortunately, during the study period, 16 patients experienced a deterioration of symptoms and unfortunately did not survive, resulting in a mortality rate of 2.28% (16/702) among pediatric patients with COVID-19 admitted to this hospital. Deceased patients, with a median age of 2 years (IQR: 1.0–2.0), had a median duration from PICU admission to death of 2 days (IQR: 1.0-3.7). Only two patients received COVID-19 vaccination and the last vaccination time was February 11, 2022 and May 11, 2023, respectively. Septic shock was the most common condition among these patients (15 cases), followed by multiple organ failure in 12 cases, and encephalopathy identified in 7 cases.

The epidemiologic and clinical characteristics in critically ill children with SARS-COV-2 Omicron infection were presented in Table 1.

**Table 1:** Demographic and clinical features of critically hospitalized children with SARS-COV-2 Omicron infection

Characteristics	Critical COVID-19 Omicron infection (*n* = 63)
**Age, years at admission (median, IQR)**	2 (1.0–8.0)
< 3	39 (61.9)
≥ 3	24 (38.1)
**Sex**	
Male	38 (60.3)
Female	25 (39.7)
**Length of PICU stay, day (median, IQR)**	14 (6.5–23.0)
Vaccination*(age ≥ 3 years)	12 (19)
One dose	3 (0.25)
Two dose	9 (0.75)
**Contact history with family members**	
Yes	30 (47.6)
Unclear	31 (52.4)
**Signs and symptoms** ^**a**^	
Fever	58 (92.1)
Fever **≥** 39 °C	45 (71.4)
Duration of fever days (median, IQR)	5 (3-8.5)
Neurological symptoms and signs ^b^	37 (58.7)
Cough	34 (54.0)
Convulsion	30 (47.6)
Dyspnea	20 (31.7)
Diarrhea	18 (28.6)
Hepatomegaly	15 (23.8)
Splenomegaly	9 (14.3)
Rash	8 (12.7)
Hematochezia	4 (6.3)
Vomiting (coffee-like substance)	4 (6.3)
Hemoptysis	3 (4.8)
Laryngeal edema	3 (4.8)
**Underlying disease**	
Epilepsy	6 (9.5)
Cerebral palsy	1 (1.6)
Nephrotic syndrome	1 (1.6)
**Complications** ^**^	
Acute respiratory failure ^c^	35 (55.6)
Encephalopathy ^d^	44 (69.8)
MIS-C ^e^	38 (60.3)
Septic shock	22 (34.9)
Multiple organs failure ^f^	11 (17.5)
Convulsion ^g^	10 (15.9)
Pneumorrhagia	5 (7.9)
Heart failure	3 (4.8)
Renal failure	2 (3.2)
Hepatic failure	2 (3.2)
Disseminated intravascular coagulation (DIC)	1 (1.6)
Severe erythema multiforme	1 (1.6)
**Oxygen supplementation**	
Mechanical ventilation	52 (82.5)
Mask oxygen inhalation/nasal prong oxygen	11 (17.5)
Duration of mechanical ventilation, day (median, IQR)	7 (3.8–13.0)
**Medical treatment**	
Antibiotic	51 (81.0)
Vasoactive drugs	40 (63.5)
Intravenous immunoglobulin	39 (61.9)
Corticosteroids	38 (60.3)
Medication for gastrointestinal diseases	18 (28.6)
Antiallergic drugs	5 (7.9)
**Outcomes**	
Improve/discharge	47 (74.6)
Death in hospital	16 (25.4)

Data are n (%) except for age, length of hospital stay and duration of fever days; IQR: interquartile range

*Children without documented receipt of any COVID-19 vaccine dose before hospitalization were considered to be unvaccinated, vaccination status was confirmed from children’s parents

^a^ Multiple presenting features were possible

^b^ Neurological symptoms and signs: includes consciousness disorders, language disorders, motor disorders, etc

^c^ Acute respiratory failure: the inability of the respiratory system to maintain oxygenation or eliminate carbon dioxide

^**^ Multiple complications were possible

^d^ Encephalopathy: a clinical state characterized by an alteration of consciousness, behaviour and/or cognition. Present with lethargy and drowsiness, or conversely with a heightened state of agitation and confusion, also can present with a range of focal neurological manifestations such as seizures, visual disturbances, speech abnormalities, motor weakness, and sensory and autonomic deficits

^e^ MIS-C: multisystem inflammatory syndrome in children

^f^ Multiple organs failure: acute lung failure, acute liver failure, acute kidney injury, cardiovascular disease, and as well as a wide spectrum of hematological abnormalities and neurological disorders

^g^ Convulsions: manifest as sudden generalized or localized muscle group tonic and clonic convulsions

### Laboratory and radiological findings

**Fig. 1:**
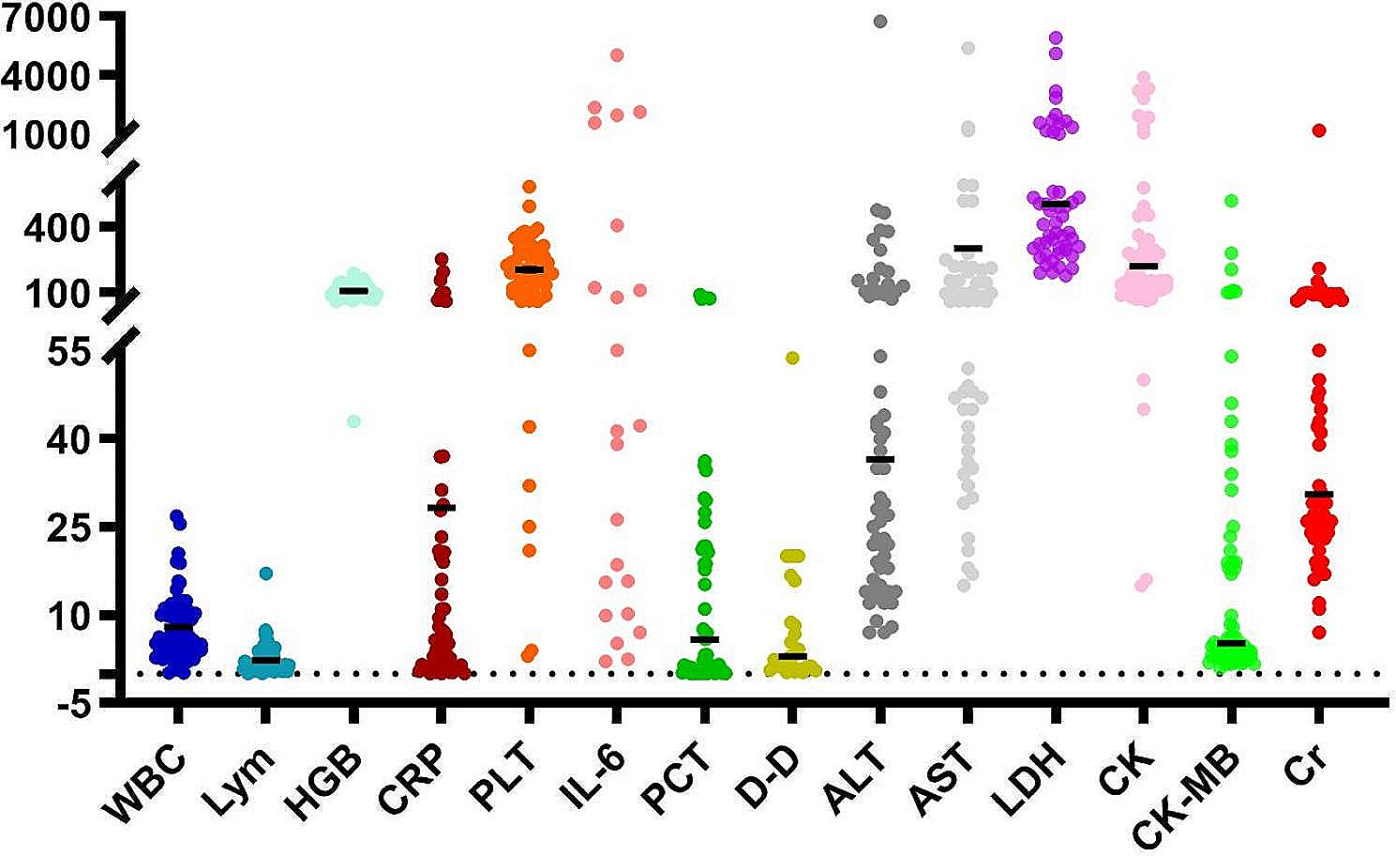
Laboratory indices among patients with SARS-CoV-2 infection

Laboratory test results revealed reduced lymphocyte counts in 58.1% (36/62) cases at admission. Elevated levels of procalcitonin (PCT), interleukin-6 (IL-6), and D-dimer were observed in 87.3% (48/55), 87% (20/23), and 88.9% (24/27) of cases, respectively. Additionally, elevated levels of alanine aminotransferase (ALT), aspartate aminotransferase (AST), lactate dehydrogenase (LDH) and creatine kinase (CK) were noted in 45.2% (28/62), 66.1% (41/62), 66.1% (41/62) and 46.8% (29/62) of cases during hospitalization, respectively (Table 2; Fig. 1).

**Table 2:** The main laboratory results and radiological findings

Laboratory finding	Critical Omicron infection (*n* = 63)
**Leucocyte (×10** ^9^ **/L)**	
Reduced	19/62 (30.6%)
Elevated	13/62 (21.0%)
**Lymphocyte (×10** ^**9**^ **/L)**	
Reduced	36/62 (58.1%)
**Hemoglobin (g/L)**	
Reduced	34/62 (54.8%)
**Platelet (×10** ^**9**^ **/L)**	
Reduced	26/62 (41.9%)
**C-reactive protein (mg/L)**	
≥ 8	28/61 (45.9%)
**Procalcitonin (ng/mL)**	
≥ 0.10	48/55 (87.3%)
**Interleukin-6 (ng/L)**	
> 7	20/23 (87%)
**D-dimer (ug/mL)**	
≥ 0.50	24/27 (88.9%)
**Alanine aminotransferase (U/L)**	
Elevated	28/62 (45.2%)
**Aspartate aminotransferase (U/L)**	
Elevated	41/62 (66.1%)
**Lactose dehydrogenase (U/L)**	
Elevated	41/62 (66.1%)
**Creatine kinase (U/L)**	
Elevated	29/62 (46.8%)
**Creatine kinase-MB (U/L)**	
Elevated	15/62 (24.2%)
**Creatinine (umol/L)**	
Elevated	19/62 (30.6%)
**Abnormal chest imaging finding**	53/56 (94.6%)
Pleural effusion	4/56 (7.1%)
Bilateral pulmonary diffuse inflammation/ Pulmonary consolidation	29/56 (51.8%)

Elevated means over the upper limit of the normal range and reduced means below the lower limit of the normal range

In terms of bacterial co-infections, 47 (75%) cases were tested for sputum bacterial.

culture and 33 of them were confirmed. The most commonly detected bacterial pathogen was *Staphylococcus aureus* (16 cases), followed by *Enterobacter species* (4 patients), *Haemophilus influenzae*, *Streptococcus pneumoniae*, and *Klebsiella pneumoniae* (3 cases each). Other bacteria detected include *Pseudomonas aeruginosa* (2 cases) and *Moraxella catarrhalis* (1 case). Additionally, one patient tested positive for *Tropheryma* *whipplei* in a sputum sample through microorganism next-generation sequencing (mNGS) (Table 3).

**Table 3:** The laboratory findings of bacterial and respiratory virus co-infection

	Bacteria and virus
**Bacterial co-infection**	**Bacterial culture (** ***n*** ** = 47)**
*Staphylococcus aureus*	16 (34)
*Enterobacter species*	4 (8.5)
*Haemophilus influenzae*	3 (6.4)
*Streptococcus pneumoniae*	3 (6.4)
*Klebsiella pneumoniae*	3 (6.4)
*Pseudomonas aeruginosa*	2 (4.3)
*Moraxella catarrhalis*	1 (2.1)
*Tropheryma whipplei*	1 (2.1)
**Respiratory virus co-infection**	**PCR assay (** ***n*** ** = 49)**
*Epstein-Barr virus*	1 (2.0)
*Adenovirus*	1 (2.0)
*Bocavirus*	1 (2.0)

Regarding respiratory virus co-infections, 8 common respiratory viruses (Flu virus A and B, parainfluenza virus, respiratory syncytial virus, rhinovirus, adenovirus, epstein-barr virus and bocavirus) were tested in throat swabs from 49 suspected cases by PCR assay. There were only 3 cases with additional respiratory viral infections (Epstein-Barr virus, Adenovirus, and Bocavirus, respectively) (Table 3).

For children with COVID-19, chest CT scans were typically used to assess the extent of pneumonia and monitor disease progression. Among the 56 patients who underwent chest CT or X-ray scans, most of the patients exhibited at least one abnormal chest imaging manifestation, such as bronchopneumonia, unilateral or bilateral pulmonary inflammation, pleural effusion, and pulmonary consolidation. Three patients showed no abnormalities on chest CT (Table 2).

Neuroimaging was indicated in cases where there were neurological symptoms or concerns, such as seizures, altered mental status, or signs of encephalopathy. A total of 37 patients underwent cranial CT or MRI examinations. Of these, 18 (49%) had no abnormalities detected within the cranium, while 19 (51%) were found to have intracranial abnormalities. Among the 19 cases, six cases were diagnosed with acute necrotizing encephalopathy (one patient had a history of epilepsy), one case was diagnosed with acute disseminated encephalomyelitis, four cases were diagnosed with encephalitis, one was diagnosed with autoimmune encephalitis, one was diagnosed with reversible posterior brain syndrome, and one case was diagnosed with reversible splenial lesion syndrome of the brain. In addition, 5 cases had other types of abnormalities (Table 4).

**Table 4:** The abnormalities of neurological manifestations in 16 patients

No.	Sex	Age	Past Medical History	Neurological Symptoms	Neuroimaging	Diagnosis	Outcome
**Case 1**	M	1y	NR	Coma; convulsion	Suspicious mild cerebral edema changes are observed in bilateral cerebral hemispheres and the brainstem.	Mild cerebral edema.	Death
**Case 2**	M	1 m	NR	Altered mental status	Abnormal signals are observed in bilateral frontal lobes and the posterior horn of the left lateral ventricle. Sequelae of hypoxic-ischemic brain injury.	Cerebral edema	Improve
**Case 3**	F	1y	NR	Convulsion	Diffuse abnormal signal changes can be observed in bilateral frontal-parietal-occipital lobes, the right temporal white matter, and the pressure part of the corpus callosum. There are also abnormal changes in the bilateral thalamus and bilateral basal ganglia.	Viral Encephalitis.	Improve
**Case 4**	F	10y	NR	Coma	Bilateral cerebral hemispheres, brainstem, and posterior thalamus show swelling and reduced density. The cerebellum is suspected to be involved.	Acute necrotizing encephalopathy.	Improve
**Case 5**	F	13y	NR	Coma; incoherent speech	Multiple scattered patchy abnormal signals are observed in the right basal ganglia region, right thalamus, brainstem, pons, and right side of the medulla oblongata. Encephalitis is being considered.	Encephalitis	Improve
**Case 6**	M	1y	NR	Coma	Both cerebral hemispheres and cerebellar hemispheres show substantial swelling. The brainstem, bilateral basal ganglia, and posterior thalamus also exhibit swelling. Acute necrotizing encephalopathy is being considered.	Acute necrotizing encephalopathy	Death
**Case 7**	F	10y	Epilepsy	Convulsion	Bilateral thalamic swelling with reduced density and pontine swelling with reduced density are observed. Acute necrotizing encephalopathy is being considered.	Acute necrotizing encephalopathy	Death
**Case 8**	F	7y	NR	Coma	Abnormal signals in the splenium of the corpus callosum are observed. Reversible splenial lesion syndrome of the brain is being considered.	Reversible splenial lesion syndrome of the brain	Improve
**Case 9**	M	17y	NR	Convulsion; coma	Bilateral temporal lobe cortical swelling with abnormal signal changes is observed. Encephalitis is being considered as a possibility.	Encephalitis	Improve
**Case 10**	M	1 m	NR	Agitation	Left temporal dura mater enhancement is observed on the MRI, with slightly increased signal in the corpus callosum.	Encephalitis	Improve
**Case 11**	M	7y	Epilepsy	Altered mental status	Abnormal signals are observed in the deep white matter area of the left frontotemporal lobe, left basal ganglia area, and left dorsal thalamus. Considering the clinical history, there is a possibility of autoimmune encephalitis.	Autoimmune encephalitis	Improve
**Case 12**	F	1y	NR	Convulsion	Bilateral thalamic swelling, brainstem edema, and multiple abnormal changes in the intracranial area are observed. Acute necrotizing encephalopathy is considered as a possibility, along with a slight hemorrhage in the left basal ganglia area.	Acute necrotizing encephalopathy	Improve
**Case 13**	M	2y6m	NR	Convulsion; coma	Swelling and decreased density in bilateral cerebral hemispheres, brainstem, and thalamus, with involvement of bilateral basal ganglia area. There is a high possibility of acute necrotizing encephalopathy.	Acute necrotizing encephalopathy.	Death
**Case 14**	F	1y	NR	Convulsion ; coma	Abnormal patchy signal changes in the bilateral thalamus, suggesting the possibility of acute necrotizing encephalitis.	Acute necrotizing encephalopathy	Improve
**Case 15**	M	4 m	NR	Convulsion	There is a significant widening of the extracerebral space and slight dilation of the ventricular system. There is also evident enhancement of the meninges in the bilateral frontal, temporal, and parietal regions, suggesting the possibility of intracranial infection.	Intracranal infection	Improve
**Case 16**	M	10y	NR	Psychomotor retardation; headache	Acute disseminated encephalomyelitis.	Acute disseminated encephalomyelitis	Improve
**Case 17**	F	2y	NR	Convulsion	Bilateral thalamic swelling, brainstem edema, and multiple abnormal changes within the cranial cavity, suggesting the possibility of acute necrotizing encephalopathy.	Acute necrotizing encephalopathy	Death
**Case 18**	M	8y	Nephrotic syndrome	Convulsion; coma	Multiple abnormal signals in the bilateral occipital and right frontal white matter, suggesting the possibility of reversible posterior brain syndrome.	Encephalopathy	Improve
**Case 19**	F	9y	NR	Convulsion	Cerebral edema.	Cerebral edema	Improve

M: male, F: female, NR: not reported. y (year), m (month)

The six cases of acute necrotizing encephalopathy presented with bilateral thalamic and bilateral basal ganglia symmetric hypodense lesions, or bilateral frontal lobe white matter hypodensity, or multiple abnormal signals within the brain. Among these cases, two were comatose within 2 days of onset, four exhibited seizures, four had a fever with a temperature ≥ 40℃, and 4 cases resulted in death (Table 4).

### Treatment and prognosis

During hospitalization, fifty-one were initially treated with antibiotics (administered orally and intravenously) which was discontinued once bacterial infection was ruled out or cured. Immunoglobulin therapy was given to 39 (61.9%) patients, while no patients received antiviral drugs therapy (Paxlovid and Remdesivir). Additionally, corticosteroid therapy was administered to 38 cases.

All patients required respiratory support, with 50 of them being placed on mechanical ventilation. The median duration of mechanical ventilation was 7 days (IQR 3.75–13.0). 16 (25%) patients experienced a deterioration in symptoms and ultimately succumbed to the illness, the main cause of death in these cases was attributed to multiple organ failure.

## Discussion

The prevalence of SARS-CoV-2 infection is particularly high among pediatric populations, especially during the Omicron variant waves. As of January 1, 2023, data from an open-access database of COVerAGE, revealed that children and adolescents under 20 years of age accounted for 21% of reported COVID-19 cases across 105 countries. Among these cases, 63% occurred in adolescents aged 10 to 19 years, while 37% occurred in children aged 0 to 9 years [18]. Evidence so far seems to indicate that intensive care admission, and deaths remained low in children with Omicron infection [19, 20]. During this outbreak, 63 children with life-threatening forms of SARS-COV-2 infection were admitted to Guangzhou Women and Children’s Medical Center, 16 deaths occurred among them.

Co-infection poses a serious complication for COVID-19, and we observed that 53 cases had bacterial co-infections, with 22 patients experiencing septic shock and 7 deaths. Bacterial co-infections among children with COVID-19 may increase mortality rates. Recent multicenter, retrospective cohort studies have reported that COVID-19 bacterial co-infections are major risk factors for mortality, ICU admission, and mechanical ventilation [21]. An observational study in Taiwan revealed that the incidence of bacterial co-infections among hospitalized children with COVID-19 was 14.9% during the Omicron pandemic [22]. Detecting co-infected pathogens is important, however, most patients with suspected secondary infections may not have undergone thorough microbiological investigations during the pandemic. In our study, the most commonly detected bacterial co-infection pathogen was *Staphylococcus aureus*, followed by *Escherichia coli*, *Haemophilus influenzae*, *Streptococcus pneumoniae*, and *Klebsiella pneumoniae*, which are similar to the common causes of bacterial infection in influenza patients [23, 24]. Laboratory test results also offer valuable insights for patients suffering from COVID-19 and concurrent bacterial co-infections. Elevated levels of PCT were observed in most patients with bacterial co-infections in our cohort. As the patients recovered, their PCT levels returned to normal, aligning with findings from other studies highlighting the predictive role of PCT in bacterial co-infection among severe and critical COVID-19 patients [25–27], although the exact association between PCT levels and co-infections or mortality in COVID-19 is not fully elucidated.

In this study, acute liver injury and myocardial damage occurred in numerous patients, characterized by abnormal biochemical parameters such as mild to significantly elevated levels of serum ALT, AST, LDH and CK. The cause is likely multifactorial, involving contributions from cytokine storm, inflammation, and drug toxicity.

While COVID-19 primarily affects the respiratory system, numerous neurological symptoms have been reported. Some symptoms, like loss of smell or taste, are mild and non-life-threatening, while others, such as seizures, are more critical [28, 29]. Neurological involvement in COVID-19 is consistently associated with worse outcomes, including ICU admission and mortality [30]. In the GCS-NeuroCOVID study, a large multicenter international study comprising over 3500 patients from three separate cohorts, 80% of the participants reported experiencing some form of neurological manifestation, with encephalopathy being the most common [31]. In the present study, we noted that 30 patients experienced encephalopathy, the cause is likely multifactorial that resulting from hypoxia–ischemia, cytokine storm, sepsis, metabolic derangement, electrolyte disturbances, medication effects, ICU delirium and a possible role for other immune mechanisms. Further studies are needed for establishing a definitive association of such symptoms with COVID-19 and also for a better comprehension of the underlying pathophysiological mechanisms.

Acute necrotizing encephalopathy (ANE), often occurs after infection with influenza virus and human herpesvirus type 6 [32, 33], but cases of ANE caused by infection with the SARS-CoV-2, especially the Omicron variant, have also been reported [34]. The typical clinical features of ANE include a short-term fever following viral infection, which then progresses rapidly to acute encephalopathy, seizures, impaired consciousness, and eventually coma. In addition to acute encephalopathy, severe cases are often accompanied by multiple organ dysfunction, disseminated intravascular coagulation (DIC), and hemophagocytic lymphohistiocytosis syndrome [35]. ANE associated with SARS-CoV-2 infection has a rapid onset and progression, often leading to death or severe neurological sequelae. Early recognition and treatment are particularly important. Febrile seizures induced by SARS-CoV-2 infection are relatively common, and some children may not exhibit typical features of simple febrile seizures [36]. In this study, 66.7% (4/6) of the ANE patients had febrile seizures, characterized by generalized tonic-clonic seizures, with 2 cases presenting as status epilepticus. In the remaining 2 cases without seizure activity, rapid onset of altered consciousness occurred, progressing quickly to coma. Therefore, when children infected with the SARS-CoV-2 present with high fever or hyperpyrexia and show neurological manifestations within a short period of time, ANE should be considered as a potential concern.

Many studies have shown that healthy children are at low risk of severe COVID-19 complications, very few children died of COVID-19 and most deaths were of children with other serious medical conditions [11–13, 18]. However, among the 16 deaths in our cohort, only two patients had underlying diseases, specifically epilepsy and acute myeloid leukemia. Most of the deaths were attributed to complications, with multiple organ failure being the primary cause. These findings suggest that even children without underlying diseases can also experience severe or critical cases of COVID-19.

This study was conducted retrospectively at a single center in China. While the sample size may not be large enough, it still provides valuable additional data on critically ill children hospitalized during the Omicron wave. The data collected in our study will contribute to enhancing our understanding of the impact of COVID-19 on children.

### Electronic supplementary material

Below is the link to the electronic supplementary material.

Supplementary Material 1

## Data Availability

Data is provided within the manuscript.
