# Supplementary material for: Critically ill children with SARS-COV-2 Omicron infection at a national children medical center, Guangdong, China
Source: BMC Pediatr. 2024 Apr 15;24:254. doi: 10.1186/s12887-024-04735-w (PMC11017605; doi:10.1186/s12887-024-04735-w)
Supplement: Supplementary file 1 — Supplementary Material 1 [file 12887_2024_4735_MOESM1_ESM.docx]

**Table 1 Comparison between patients with and without neurological complications**

| **Characteristics** | **Patients with neurological complications**  **(n=19)** | **Patients without neurological**  **complications**  **(n=44)** | ***P-*value** |
| --- | --- | --- | --- |
| **Age, years at admission (median, IQR)** | 2.5 (1.0-10.0) | 2.0 (0.6-5.7) | 0.198 |
| 0-3y | 10 (52.6) | 32 (72.7) | 0.151 |
| >3y | 9 (47.4) | 12 (27.3) |  |
| **Sex** |  |  |  |
| Male | 10 (61.1) | 28 (63.6) | 0.575 |
| Female | 9 (38.9) | 16 (36.4) |  |
| **Length of PICU stay, day (median, IQR)** ^†^ | 15 (5.0-22.0) | 13 (9.3-24.3) | 0.331 |
| **Vaccination*（age≥3 years)** | 5/9 (55.6) | 7/18 (38.9) | 0.683 |
| **Fever** | 19 (100.0) | 44 (100.0) | - |
| Peak temperature ≥39℃ | 11 (57.9) | 34 (77.3) | 0.138 |
| Duration of fever days (median, IQR) | 4 (6.0-23.0) | 16 (6.0-23.0) | 0.258 |
| **MIS-C**^▲^ | 13 (68.4) | 25 (56.8) | 0.418 |
| **Outcome** |  |  |  |
| Cure/discharge | 14 (73.7) | 33 (75.0) | >0.999 |
| Death (in-hospital mortality) | 5 (26.3) | 11 (25.0) |  |
| **Laboratory finding** |  |  |  |
| Leucocyte (×10^9^/L) |  |  |  |
| Reduced | 2/18 (11.1) | 17/44 (38.6) | 0.038 |
| Elevated | 2/18 (11.1) | 10/44 (22.7) | 0.481 |
| Lymphocyte (×10^9^/L) |  |  |  |
| Reduced | 10/18 (55.6) | 26/44 (59.1) | >0.999 |
| C-reactive protein (mg/L) |  |  |  |
| ≥8 | 16/18 (33.3) | 22/24 (91.7) | >0.999 |
| Procalcitonin (ng/mL) |  |  |  |
| ≥0.10 | 16/17 (94.1) | 31/37 (83.8) | 0.412 |
| Interleukin-6 (ng/L) |  |  |  |
| >7 | 3/4 (75.0) | 17/19 (89.5) | >0.999 |

Data are n (%) except for age, length of PICU stay and duration of fever days; IQR: interquartile range.

^†^ Excluding death patients.

^*^ Children without documented receipt of any COVID-19 vaccine dose before hospitalization were considered to be unvaccinated, vaccination status was confirmed from children’s parents.

^▲^ MIS-C: multisystem inflammatory syndrome in children.
